# Protocol for a primary human 2D intestinal epithelium-dendritic cell co-culture organoid model to study mucosal immunity and viral infections

**DOI:** 10.1016/j.xpro.2026.104740

**Published:** 2026-08-04

**Authors:** Anusca Germaine Rader, Carla Mónica Sampaio Ribeiro

**Affiliations:** 1 Amsterdam UMC location University of Amsterdam, Center for Infection and Molecular Medicine, Amsterdam, the Netherlands; 2 Amsterdam institute for Immunology & Infectious Diseases, Amsterdam, the Netherlands; 3 Amsterdam Gastroenterology Endocrinology and Metabolism, Amsterdam, the Netherlands

**Keywords:** Immunology, Microbiology, Microscopy, Organoids

## Abstract

The intestinal mucosa is a major HIV-1 infection site, making immune-augmented intestinal models essential for mechanistic and therapeutic studies. We present a protocol that converts human intestinal organoids into polarized 2D epithelial monolayers and incorporates basolateral dendritic cells to create a biomimetic intestinal mucosal model. We describe steps for monolayer formation, integrity assessment, immune cell co-culture, virus inoculation, and analyses by flow cytometry and confocal microscopy. This immuno-organoid platform enables controlled HIV-1 exposure with immunological and virological readouts.

For complete details on the use and execution of this protocol, please refer to Rader et al.^1^

## Highlights

• Steps for generating polarized 2D epithelial monolayers from 3D intestinal organoids
• Instructions for co-culturing epithelial monolayers with basolateral dendritic cells
• Guidance on immunophenotyping and functional assessment of host-virus interactions

## Abstract (editor highlights)

Publisher’s note: Undertaking any experimental protocol requires adherence to local institutional guidelines for laboratory safety and ethics.

## Graphical abstract

**Figure:**
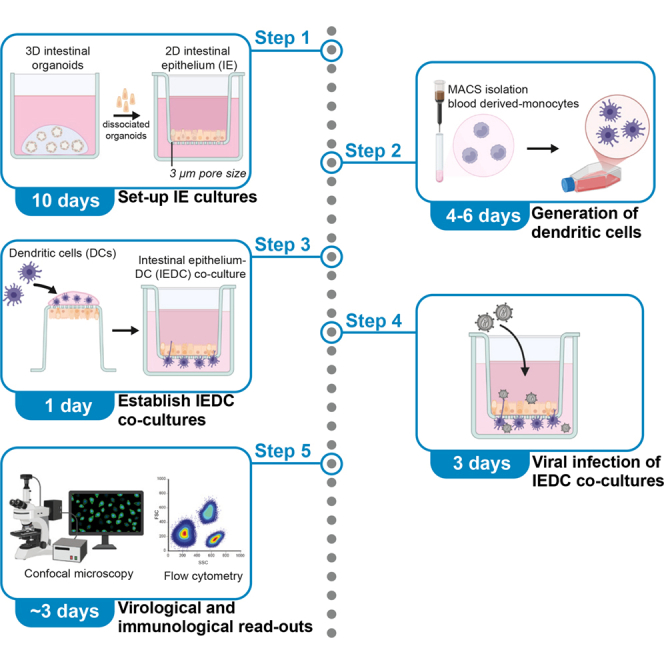

## Before you begin

The intestinal mucosa is recognized as a primary site for HIV-1 acquisition, early dissemination, and the establishment of viral latency. Dendritic cells (DCs) in the human intestinal lamina propria act as a critical cellular niche that supports HIV-1 disease, contributing to both the rapid viral dissemination during early stages of infection and the establishment of viral reservoirs.^2^^,^^3^ Understanding mechanisms of HIV-1 infection across the intestinal mucosa and epithelial-dendritic cell interactions is therefore important for developing effective preventative and curative strategies. This protocol describes the specific steps to establish polarized 2D intestinal epithelial monolayers cultured on collagen-coated cell culture inserts derived from 3D human intestinal organoids. We detail quality control assessments of epithelial barrier integrity by transepithelial electrical resistance (TEER) and paracellular permeability analyses. Next, we describe the differentiation of monocyte-derived dendritic cells from peripheral blood mononuclear cells (PBMCs) and their subsequent integration onto the basolateral side of epithelial monolayers to generate intestinal epithelium-DC co-cultures. We then provide detailed instructions for experimental HIV-1 infection of these 2D immuno-organoids and the applicability of the subsequent immunological and virological readouts for comprehensive analyses of host-virus interactions and host immune responses of enteric viral diseases in vitro.

### Innovation

This protocol establishes human-centric intestinal epithelium-DC co-cultures to investigate mucosal HIV-1 disease. Prior in vitro intestinal co-culture models have largely relied on immortalized epithelial cell lines such as Caco-2, which do not recapitulate the diversity and distinct functions of intestinal epithelial cell types in vivo.^4^^,^^5^ The central novelty of our work is combining polarized 2D epithelial monolayers derived from primary human fetal intestinal organoids with primary monocyte-derived DCs positioned onto the basolateral surface of the cell-culture insert.^1^ The 2D configuration also offers distinct advantages over enclosed 3D organoid models: it preserves epithelial polarity and barrier integrity while enabling compartmentalized analysis of apical and basolateral microenvironments independently.^6^ Critically, the 2D format allows continuous monitoring of epithelial barrier function, which is difficult to obtain in 3D systems.^7^^,^^8^ In addition, the stable epithelial-DC contact at the basolateral surface ensures physiologically relevant immune positioning while maintaining direct apical access for controlled, luminal or basolateral viral exposure. This model allows the evaluation of immunovirological readouts that capture both immune compartment functionality and viral infection dynamics, including confocal microscopy to resolve spatial virus-host (sub)cellular interactions and flow cytometry for immunophenotyping of dendritic cells. Altogether, this platform provides a biomimetic intestinal mucosal model to interrogate primary epithelial barrier dynamics, mucosal immunity, and HIV-1 pathogenesis in a scalable in vitro system.

### Institutional permissions

Prior approval for the use of human fetal material and buffy coats is required by your local ethics committee. Here, human fetal intestinal tissues used to generate organoids were obtained from the HIS Mouse Facility of the Amsterdam UMC, The Netherlands. All material has been collected from anonymized donors from whom a written informed consent has been obtained for the use of the material for research purposes. The use of the anonymized material for medical research purposes is covered by a Dutch law (Wet foetaal weefsel and Article 467 of Behandelingsovereenkomst). Tissues were obtained with approval of the experimental procedures by the HIS Mouse Facility (Amsterdam UMC). Buffy coats derived from blood donations (Sanquin blood bank, the Netherlands) were obtained with approval of the Medical Ethics Review Committee of the Amsterdam UMC. Buffy coats were handled in accordance with the relevant guidelines and regulations, as stated in the Amsterdam UMC Research Code. Use of buffy coats is not subjected to informed consent according to the Medical Research Involving Human Subjects Act and the Medical Ethics Review Committee of Amsterdam UMC. All HIV-1 related work was conducted in Biosafety safety level-3 containment level at Amsterdam UMC with institutional permission for this research. Users must obtain local training certifications and institutional approvals before working with infectious HIV-1 strains.

### Preparation of collagen-coated cell-culture inserts

**Timing: 2 h**

**CRITICAL:** Acetic acid is corrosive. Wear gloves when handling the solution. In addition, the preparation of the collagen-coated cell culture inserts should be performed in biological safety cabinets under sterile working conditions.

***Note:*** Collagen is insoluble at a neutral pH, it is therefore diluted in 0.1% acetic acid to maintain it in solution.

1. Prepare 0.1% acetic acid solution at room temperature (RT).
  a. Add 10 μL acetic acid to 10 mL distilled water in a 50 mL conical tube and invert a few times to mix (volume can be adjusted to the amount of solution needed).
  b. Filter 0.1% acetic acid solution through a 0.45 μm filter before use.

***Note:*** A 0.22 μm filter may be used for stricter sterile filtration without changing the subsequent steps.

2. Prepare 20 μg/mL collagen type I coating solution.
  a. Dissolve 40 μL rat collagen type I in 10 mL 0.1% acetic acid solution at RT.
3. Place 3.0 μm pore 24-well cell culture inserts in a 24-well plate and coat by adding 100 μL coating solution into the apical side of the insert.

***Note:*** The 3.0 μm pore size was chosen to enable DC luminal sampling and protrusions toward the epithelium, while still supporting epithelial monolayer formation.

4. Incubate for 1 h at RT.
5. Remove coating solution and wash the inserts twice with 200 μL sterile PBS at RT.

***Note:*** Remove all PBS by first pipetting out PBS with 200 μL pipet followed by removing final volume with a 20 μL pipet.

6. Let inserts dry completely in the biological safety cabinet at RT for approximately 5-10 min before use for experiments or storage until use.

**Figure 1:**
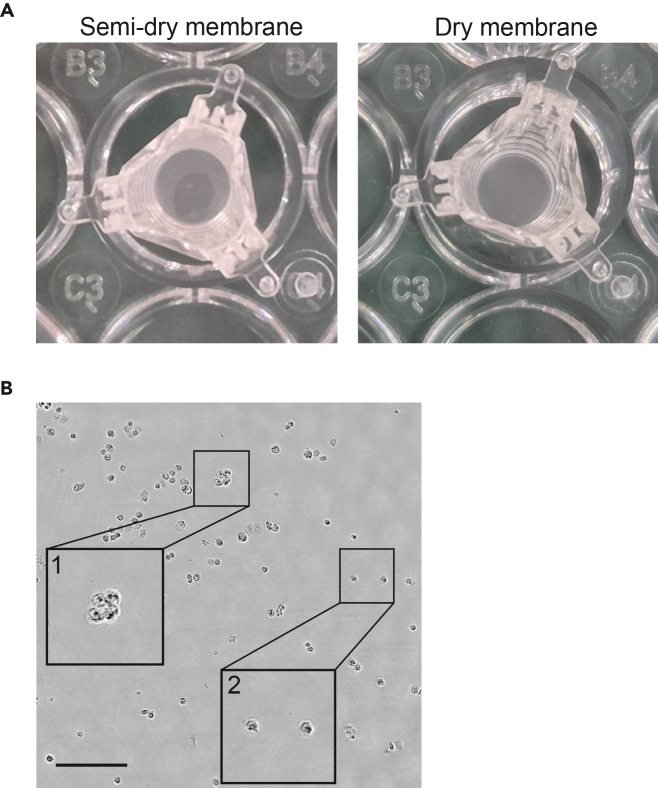
Drying of collagen-coated inserts and visualization of dissociated intestinal epithelial cells for monolayer seeding (A) Representative images of semi-dry and dry insert membranes after collagen coating. (B) Intestinal epithelial cells after TrypLE-based dissociation of 3D organoids, illustrating the desired cell clusters (inset 1) and single-cell state (inset 2) for appropriate 2D epithelial monolayer seeding. Images acquired using the Incucyte S3. Scale bar = 200 μm.

***Note:*** The inserts dry faster with the 24-well plate lid open in a biological safety cabinet. The insert membranes will appear white when dry (Figure 1A).

***Note:*** When storing coating inserts for later use, the plates with inserts should be sealed (for example, wrapped tightly in plastic film such as parafilm or kitchen cling film) to prevent contamination. Coated inserts can be stored at 4°C for 4 weeks.

### Preparation of saponin-based cell permeabilization buffer for flow cytometry

**Timing: 10 min**

7. Dissolve saponin to a final concentration of 0.5% (m/v) and bovine serum albumin (BSA) to 1% (m/v) in PBS mL in a conical tube.
8. Place the conical tube on an automated roller at medium speed for 5 min at RT, or until the saponin and BSA have dissolved.
9. Store at 4°C for up to 7 days.

### Preparation of 0.5% Triton X-100 permeabilization buffer for confocal microscopy

**Timing: 35 min**

10. Add 50 mL PBS to a conical tube.
11. Add Triton X-100 to a final concentration of 0.5% (v/v).

**CRITICAL:** Triton X-100 is very viscous, making precise pipetting challenging. Use wide-bore tips or cut of the end of a 1mL tip to correctly pipet the pre-defined volume.

12. Mix on an automated roller at medium speed for 30 min at RT, or until Triton X-100 has dissolved.
13. Store at RT.

## Key resources table

**Table:** 

REAGENT or RESOURCE	SOURCE	IDENTIFIER
**Antibodies**		

Mouse IgG1 anti-CD45-BV711 (clone HI30, dilution 1:100)	Biolegend	304049
Mouse IgG1 anti-CD11c-PE-Cy7 (clone B-ly6, dilution 1:50)	BD Biosciences	561356
Mouse IgG2a anti-DC-SIGN-AF750 (clone 120612, dilution 1:100)	Biotechne	1719206
Mouse IgG1 anti-p24 (clone KC57-PE, Dilution 1:100)	Beckman	KC57-RD1
Mouse IgG1 anti-CD83-AF647 (clone HB15e, dilution 1:50)	Biolegend	305316
Mouse IgG2b anti-CD86-AF488 (clone IT2.2, dilution 1:50)	Biolegend	305414
Mouse IgG2b anti-CD11c-PE (clone S-HCL-3, dilution 1:20)	BD biosciences	333149
Goat anti-mouse IgG2b AF546 (dilution 1:400)	Invitrogen	A-21143
Goat anti-mouse IgG1 AF647 (dilution 1:400)	Invitrogen	A-21240

**Biological samples**		

Primary human intestinal epithelial cells	Isolated from human fetal intestinal tissue samples obtained from the HIS Mouse facility of the Amsterdam UMC with the required institutional permissions.	N/A
Peripheral blood mononuclear cells	Isolated from buffy coats of healthy donors acquired through Sanquin with the required institutional permissions.	N/A

**Chemicals, peptides, and recombinant proteins**		

Phosphate Buffered Saline (PBS; 1x)	Gibco	10010023
Dulbecco’s Phosphate Buffered Saline (DPBS; 1x)	Lonza	17-512F
Ethylenediaminetetraacetic acid solution (EDTA)	Sigma-Aldrich	03690-100ML
Advanced DMEM/F12	Thermo Fisher Scientific	12634010
GlutaMAX Supplement	Invitrogen	35050061
HEPES	Sigma Aldrich	H3375
Penicillin/Streptomycin	Thermo Fisher Scientific	15140122
IntestiCult OGM Human Basal Medium	STEMCELL Technologies	100-0190
Organoid Supplement	STEMCELL Technologies	100-0191
IntestiCult ODM Human Basal Medium	STEMCELL Technologies	100-0212
DAPT	STEMCELL Technologies	100-1046
RPMI 1640, no glutamine	Gibco	21870076
L-glutamine	Lonza	BEBP17-605E
IL-4	Invitrogen	A42603
GM-CSF	Invitrogen	BMS324
Fetal Bovine Serum (FBS)	TRINITY TEK	01010102
Paraformaldehyde, 8% w/v aq. soln., methanol free	Thermo scientific	047347.9M
Saponin	Sigma Aldrich	SAE0073
Bovine Serum Albumin (BSA), Fraction V	Roche	10738328103
Triton X-100	Sigma Aldrich	X100
ROCK inhibitor Y-27632	STEMCELL Technologies	72302
Collagen type I	Ibidi	50202
TrypLE Express Enzyme (1x)	Gibco	12605010
Lymphoprep	Axis-Shield	AXS-1114544-AXS-111575
Heparin	Leo Pharmaceuticals	PL 00043/0038R
4 kDa FITC-conjugated dextran	Sigma Aldrich	FD4-100MG
Hanks’ Balanced Salt Solution without phenol-red	Gibco	12549069
Accutase Cell Detachment Solution	STEMCELL Technologies	07920
DAPI	Invitrogen	D1306
Prolong Gold Diamond Antifade mountant	Invitrogen	P36965
Acetic acid	Sigma Aldrich	695092
Fish serum blocking buffer	Thermo Fisher Scientific	37527
Phalloidin CruzFluor-488 (dilution 1:1500)	Santa Cruz Biotechnology	sc-363791

**Critical commercial assays**		

CD14+ Microbeads	Miltenyi	130-050-201

**Software and algorithms**		

Leica application Suite X	Leica microsystems	
FlowJo version 10.6.2	Flowjo, LLC	
GraphPad Prism 9	Graphpad Software Inc.	

**Other**		

Black flat-bottom 96-well polystyrene plates	Corning	CLS3915
24-well cell culture inserts PET, 3.0 μm clear	SABEU - CellQart	9323012
Corning Falcon Centrifuge tubes, 50 mL	Corning	CLS352070
Falcon 15 mL Conical Centrifuge Tubes	Falcon	14-959-53A
Eppendorf tubed 3810 - Microtube	Eppendorf	0030125150
Corning Costar TC-treated multiple wells plates, 24-well	Corning	CLS3527-100EA
Cell culture multiwell plate, 6 well	Greiner	657160
Millicell ERS 3.0 Digital Voltohmmeter	Millipore	MERS03000
Millicell ERS 3.0 Standard Electrode	Millipore	MERS03SAP
Scalpel blades No.11	Romed HOLLAND	BLADE11
Scalpel blades No.10	Romed HOLLAND	BLADE10
Scalpel handle number 3	Romed HOLLAND	HANDLE-3
Thermo Scientific™ Microscope Slides, Cut, 1mm	Thermo scientific	12342108
Marienfeld Superior X2000 Precision cover glasses thickness no. 1.5H (0.17 mm ± 0.005 mm), 18x18 mm, 10x200 pcs./carton	Marienfeld Superior	1590396

## Materials and equipment

**Table:** MACS buffer

Reagent	Final concentration	Amount
PBS	N/A	448 mL
EDTA (0.5M)	2mM	2 mL
FBS	1%	50 mL
**Total**	**N/A**	**500 mL**

Store at 4°C and use within 3 months.

**Table:** Monocyte-derived dendritic cell (moDC) culture medium

Reagent	Final concentration	Amount
RPMI 1640	N/A	440 mL
FBS	10%	50 mL
Penicillin/Streptomycin (100x)	1x	5 mL
L-glutamine (200mM)	2mM	5 mL
IL-4 (2x10^6^ U/mL)^a^	500 U/mL	125 μL
GM-CSF (4x10^6^ U/mL)^a^	800 U/mL	100 μL
**Total**	**N/A**	**500 mL**

a Cytokines should be aliquoted and stored in −20 °C to reduce freeze-thaw cycles, and need to be added fresh just before starting the moDC cultures.

All components required for moDC culture medium (except for the cytokines IL-4 and GM-CSF) can be mixed upfront and stored at 4°C for up to 3 months.

**Table:** Advanced+++ culture medium

Reagent	Final concentration	Amount
Advanced DMEM/F12	N/A	485 mL
GlutaMAX (100x)	1x	5 mL
HEPES (1M)	10 mM	5 mL
Penicillin/Streptomycin (100x)	1x	5 mL
**Total**	**N/A**	**500 mL**

Store at 4°C.

**Table:** Intestinal organoid expansion medium

Reagent	Final concentration	Amount
IntestiCult OGM Human Basal Medium	N/A	10 mL
Organoid Supplement	N/A	10 mL
Penicillin/Streptomycin (100x)	1x (10 U/mL and 10 μg/mL, respectively)	200 μL
**Total**	**N/A**	**20 mL**

To reduce freeze-thaw cycles of IntestiCult OGM Human Basal Medium and Organoid Supplement, aliquot the media into 5-10 ml portions. Make the amount required for culturing (e.g., for 10 culture inserts prepare 6 mL). Store at 4°C and use within 7 days.

**Table:** Intestinal organoid differentiation medium

Reagent	Final concentration	Amount
IntestiCult ODM Human Basal Medium	N/A	10 mL
Organoid Supplement	N/A	10 mL
Penicillin/Streptomycin (100x)	1x	200 μL
DAPT (5mM)	5 μM	20 μL
**Total**	**N/A**	**20 mL**

To reduce freeze-thaw cycles of IntestiCult ODM Human Basal Medium, aliquot the medium into 5-10 ml portions. Make the amount required for culturing (e.g., for 10 culture inserts prepare 6 mL). Store at 4°C and use within 7 days.

**Table:** Intestinal epithelium-DC (IEDC) co-culture medium

Reagent	Final concentration	Amount
IntestiCult ODM Human Basal Medium	N/A	10 mL
Organoid Supplement	N/A	10 mL
Penicillin/Streptomycin (100x)	1x	200 μL
DAPT (5mM)	5 μM	20 μL
GM-CSF (4x10^6^ U/mL)	800 U/mL	4 μL
**Total**	**N/A**	**20 mL**

Make fresh before use.

**Table:** 4% paraformaldehyde solution

Reagent	Final concentration	Amount
Paraformaldehyde, 8% w/v aq. soln., methanol free	4%	10 mL
PBS	N/A	10 mL
**Total**	**N/A**	**20 mL**

The 4% solution is stable up to 7 days at 4°C. Protect from light.

**CRITICAL:** Paraformaldehyde is toxic and releases vapors, handle in a fume hood with gloves and dispose of waste according to institutional safety regulations.

**Table:** Cell permeabilization buffer for flow cytometry

Reagent	Final concentration	Amount
PBS	N/A	50 mL
Saponin	0.5%	0.25 g
BSA	1%	0.5 g
**Total**	**N/A**	**50 mL**

Store at 4°C and use within 7 days.

**CRITICAL:** Saponin is an irritant and it is advised to wear gloves when handling the buffer, avoid inhalation of the dry powder.

**Table:** 0,5% Triton X-100 for confocal microscopy

Reagent	Final concentration	Amount
Triton X-100 (100%)	0.5%	250 μL
PBS	N/A	50 mL
**Total**	**N/A**	**50 mL**

Store at RT for up to 6 months.

**CRITICAL:** Triton X-100 is an irritant and environmentally hazardous, wear gloves when handling and dispose of waste according to institutional safety regulations.

## Step-by-step method details

### Establishment of polarized 2D intestinal epithelial monolayers on collagen-coated cell-culture inserts

**Timing: 10 days total, 1 h hands-on on days 0, 3, 5, 7, and 10**

**Figure 2:**
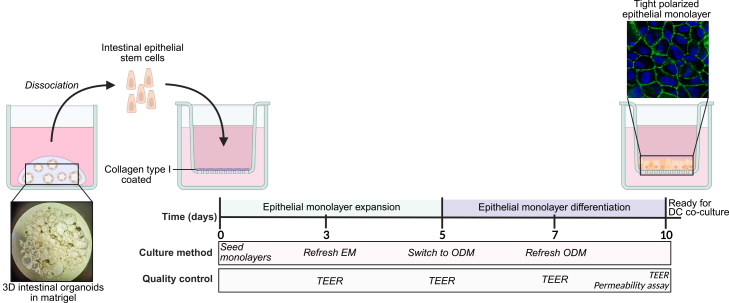
Establishment of a primary human intestinal epithelium culture model Schematic representation and timeline of the establishment and quality control of a confluent primary human intestinal epithelium culture model. 3D intestinal organoids were dissociated into single cells, seeded on collagen type I-coated cell culture inserts, expanded in expansion medium (EM), and differentiated in organoid differentiation medium (ODM) to form tight, polarized intestinal epithelial monolayers after. Barrier integrity was monitored throughout culture using transepithelial electrical resistance (TEER) measurements, and 4 kDa FITC-dextran paracellular permeability assays were performed at the end of the culture period prior to co-culture.

In this step, we detail the generation of 2D intestinal epithelial (IE) monolayers on cell-culture inserts from 3D intestinal organoids (Figure 2).

***Note:*** The description for the generation of 3D intestinal organoids from human fetal tissue can be found in the protocol by Schreurs et al.^9^ Please refer to that publication for detailed steps on stem cell isolation and 3D organoid establishment and culturing.

***Note:*** 3D intestinal organoids cultured in expansion medium are ready for 2D IE monolayer formation when they become cystic (spherical structures with a central lumen, Figure 2). This is after at least 3 passages after tissue isolation or after thawing organoids from cryopreservation, depending on the donor. For organoid densities comparable to those shown in Figure 2, one well of 3D organoids generally yield sufficient cells to seed 3 monolayers inserts (see troubleshooting 1).

***Note:*** If possible, culture additional IE monolayers specifically for the quality control assays mentioned in the text section to account for increased potential of barrier disruption due to repeated handling.

1. Dissociate 3D intestinal organoids to obtain a single cell suspension.
  a. Add ice-cold 1mL Advanced+++ to 15 mL conical tubes, 1 tube per donor.
  b. Harvest organoids from each well in 800 μL ice-cold Advanced+++ and resuspend. For each donor, pool organoids from all wells into one labeled 15 mL conical tube.
  c. Spin down 5 min at 140 x g at 4°C and remove supernatant.
    i. Carefully aspirate and discard the cloudy supernatant, which contains the Matrigel in which the 3D organoids were previously embedded.
  d. Resuspend organoids in 0.5 mL pre-warmed TrypLE per 1-3 wells of organoids (total volume 0.5–2 mL depending on the number of wells harvested) and incubate conical tubes horizontally at 37°C in a humidified incubator at 5% CO_2_ for 10 min.
  e. Pipette the suspension 10-20 times up and down with a p200 pipette to mechanically dissociate the organoids into single cells.
  f. Check dissociation of the organoids under the microscope by holding the conical tube on the microscope stage.
    ***Note:*** Formation of IE monolayers is more efficient when organoids are disrupted into smaller clusters of 2-4 cells rather than only into individual cells (Figure 1B). If needed, adjust the TrypLE digestion time within a range of 5-15 min total to achieve the desired cluster size.
  g. Fill the conical tube to 15 mL with room temperature Advanced+++ supplemented with 15% FBS to block the digestion.
  h. Count cells with a hemocytometer or automated cell counter.
  i. Spin down cells for 5 min at 320 x g at RT.
2. Resuspend cells in Intestinal organoid expansion medium (EM) supplemented with 10 μM Y-27632 to a final concentration of 1x10^6^ cells/mL.
  a. Example, for 5×10^5^ cells, resuspend the pellet in 0.5 mL of EM supplemented with 10 μM Y-27632.
3. Add 100 μL of cell suspension (=100.000 cells per insert) to the apical side of the collagen-coated cell culture insert and pipette once up and down to distribute the cells evenly.

**CRITICAL:** Make sure to avoid generating bubbles during pipetting by dispensing slowly to the first stop only, as bubble formation can negatively affect monolayer formation by preventing local cell adhesion and causing an uneven distribution of cells across the insert.

4. Add 500 μL EM supplemented with Y-27632 to the basolateral side and culture the cells in 37°C in a humidified incubator at 5% CO_2_ (see troubleshooting 2).
5. After 3 days of culture, first remove the basolateral and then the apical medium and gently refresh both with pre-warmed EM without Y-27632, using the same volumes as during initial culturing.
6. After 5 days of culture, refresh to Intestinal organoid differentiation medium (ODM) to induce differentiation of the monolayers.
7. After 7 days of culture, refresh the ODM.
8. After 10 days of culture, refresh the ODM and the polarized differentiated 2D monolayers are now ready for downstream applications.

### Assessment of intestinal epithelial barrier integrity

**Timing: 30 min to 5 h**

**Figure 3:**
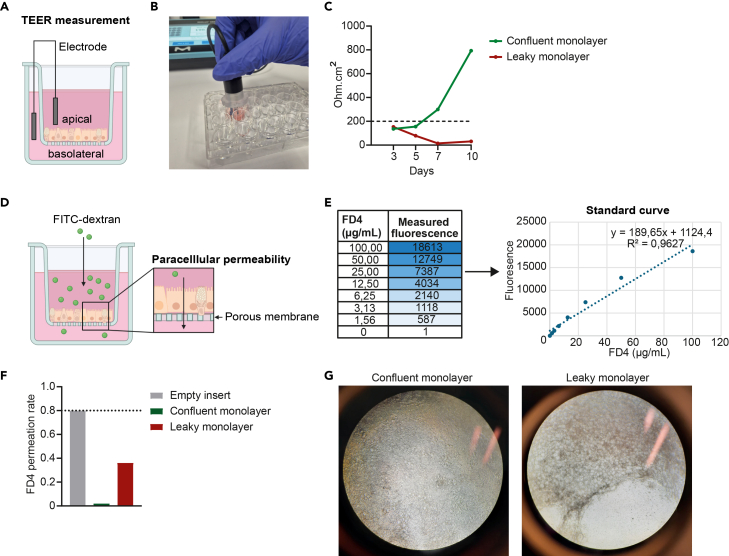
Validation of intestinal epithelial barrier integrity (A) Schematic depiction of electrode positioning on the intestinal epithelium culture model to measure transepithelial electrical resistance (TEER). (B) Photograph of the TEER measurement. (C) Data of longitudinal TEER measurements at days 3, 5, 7, and 10 for both confluent and leaky monolayers. Dashed line at 200 Ohm.cm^2^ indicates confluency. (D) Schematic depiction of 4 kDa FITC-dextran (FD4) paracellular transport through the epithelial barrier model. (E) Standard curve relating measured fluorescence to FD4 concentration. (F) FD4 permeability rates for empty inserts, confluent monolayers, and leaky monolayers. Dotted line indicates the permeability of empty inserts. Permeability is expressed as FD4 permeation rate: FD4 basolateral _t = 4 h (μg/mL)_/FD4 apical _t = 0 h (μg/mL)._ (G) Representative pictures of confluent and leaky epithelial monolayers as seen through the ocular of an inverted microscope at 10x magnification.

To ensure the establishment of a tight polarized epithelial monolayer, epithelial barrier integrity is monitored by longitudinal transepithelial electrical resistance (TEER) measurements using a Volt- ohmmeter on medium refreshment days (Figures 3A–3C), and by a paracellular permeability assay using fluorescent tracer flux after 10 days of culture (Figures 3D–3F). Together, these assays confirm that epithelial barrier integrity is established before proceeding with downstream applications (Figure 3G).

9. Transepithelial electrical resistance measurement.
  a. Refresh medium in cell culture inserts with pre-warmed EM or ODM.
  b. Electrode cleaning before measurement.
    i. Label three 50 mL conical tubes as EtOH (70%), H_2_O, and PBS, and fill each tube with ∼40 mL of the respective solution.
    ii. In the flow cabinet, rinse the electrode sequentially in 70% EtOH, H_2_O, and PBS for approximately 5 seconds in each solution prior to use and between different intestinal donors.
      ***Note:*** TEER measurements should be done on the day of media refreshing.
      ***Note:*** Include an empty cell culture insert as a background control (expected resistance: ∼100–200 Ω).
  c. Switch to “Ω” mode on Epithelial Volt/Ohm meter (EVOM2).
  d. To measure TEER, position the long electrode tip just outside the cell culture insert (so inside the well), and the short tip just above the membrane inside the insert (Figures 3A and 3B).
  e. Measure TEER three times per insert, sampling across all three open positions of the insert. Allow readings to stabilize and record the average value.
    **CRITICAL:** Perform TEER measurement at RT under consistent temperature conditions across all time points to minimize fluctuations, ideally 20 min after medium refreshment to allow the cultures to equilibrate to the new medium.
  f. To clean the electrode post-measurement. Wash the electrode in EtOH and H_2_O for approximately 5 seconds in each solution, and allow to air dry or gently wipe with tissue.
  g. Calculate TEER values (Ω·cm^2^) by subtracting the background resistance of the empty insert and multiplying by the surface area (0.32mm^2^) of the insert membrane.
    ***Note:*** Monolayers were considered sufficiently formed when TEER values exceeded 200 Ω·cm^2^ (Figure 3C), indicating the establishment of a tight epithelial barrier (see troubleshooting 3 and 4).
10. Paracellular permeability assay.
  a. Wash monolayers with HBSS without phenol red at RT by briefly adding and aspirating the buffer.
    ***Note:*** Include an empty cell culture insert as control.
  b. Add 110 μL of HBSS containing 100 μg/mL 4 kD FITC-Dextran (FD4) to the apical side.
  c. Add 700 μL HBSS to the basolateral side.
  d. Collect t = 0 h samples.
    i. 10 μL from the apical side and 100 μL from the basolateral side.
  e. Transfer samples to a black, flat-bottom 96-well plate, protect from light, and store at 4 °C.
    i. Top up all wells in the black plate with HBSS to a final volume of 100 μL.
  f. Incubate cultures for 4 h at 37 °C, 5% CO_2_, in a humidified incubator.
  g. Collect t = 4 h samples (as step 10d-e).
    ***Note:*** Collected FD4 samples can be stored up to 72 h in 4 °C without significant loss of signal.
  h. Prepare a FD4 standard curve (100, 50, 25, 12.5, 6.25, 3.13, 1.56, 0 μg/mL).
  i. Measure fluorescence using a plate reader with excitation and emission filters 485:20 nm and 528:20 nm, respectively.
  j. Determine FD4 concentrations using the standard curve (Figure 3E).
  k. Calculate the FD4 permeation rate using the following equation: $\frac{{\text{FD}4\;\text{basolateral}}_{t=4\;h\left(\mu g/\text{mL}\right)}}{{\text{FD}4\;\text{apical}}_{t=0\;h\left(\mu g/\text{mL}\right)}}$. Here, μg/mL indicates the amount of FD4 calculated from the fluorescence measurements described in the previous steps (Figure 3F).
    **CRITICAL:** Paracellular permeability assays cannot be applied to IEDC co-cultures, as dendritic cells phagocytose the dextran molecules. However, TEER measurements can still be used to assess barrier integrity of the IEDC co-cultures.

### Isolation and differentiation of monocytes to immature dendritic cells

**Timing: 3 h, 4 days of incubation**

In this step, monocytes are isolated from buffy coats to be differentiated into monocyte-derived dendritic cells.

***Note:*** This protocol is an adjusted version of a previously described protocol.^10^^,^^11^ The protocol here describes the isolation from one donor. For the simultaneous isolation of multiple donors, multiply the materials used accordingly.

***Note:*** One buffy coat should provide 400-900∗10^6^ PBMCs.

**Figure 4:**
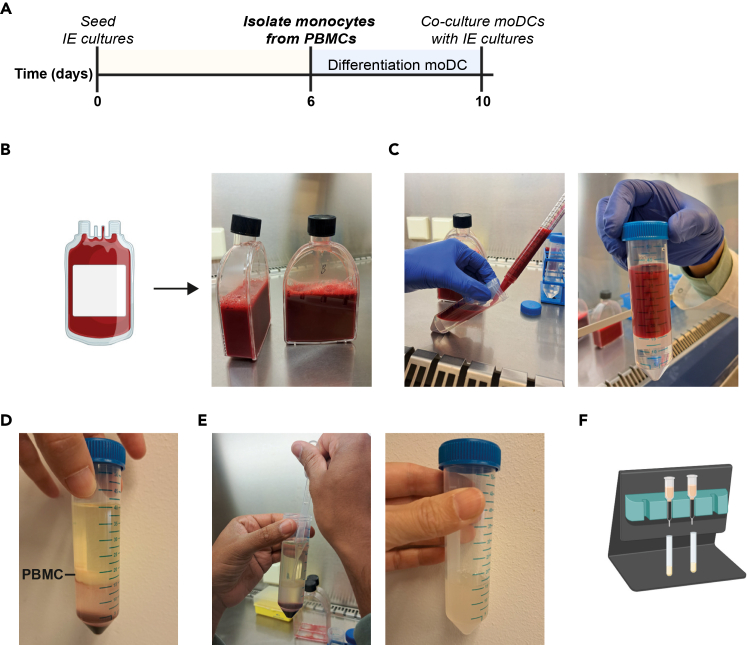
PBMC isolation from buffy coats (A) Timeline indicating initiation of monocyte isolation and moDC differentiation suitable for co-culture with intestinal epithelium (IE) cultures. (B) Buffy coat is diluted in heparin PBS solution in a T75 flask. (C) Diluted buffy coats are carefully layered on top of the Lymphoprep layer. (D) After centrifugation, the PBMC interphase is visible. (E) The PBMC interphase is collected using a Pasteur pipette. (F) Washed PBMCs are ready for magnetic-activated cell sorting (MACS) of CD14+ monocytes.

**CRITICAL:** Ensure that monocyte-derived dendritic cells are ready to be added to 10-days-old IE cultures to optimally generate IEDC co-cultures (Figure 4A). We typically use 2D monolayers from day 10 onward, when they form a confluent polarized monolayer as per quality control assays. Dendritic cells can also be attached at later time points, but in our hands the 2D monolayers remain stable for approximately 15 days in total.

11. Prepare heparin PBS solution.
  a. Add 30 mL of PBS at RT to a T75 cell culture flask.
  b. Add 300 μL of heparin to the PBS in the flask.
12. Dilute Buffy Coat (see troubleshooting 5).
  a. Transfer the entire volume of the buffy coat into the T75 flask containing the heparinized PBS (Figure 4B).
  b. Adjust the total volume to 150 mL using PBS (RT).
13. Prepare density gradient solution.
  a. Label six 50 mL conical tubes using a marker.
  b. Add 15 mL of Lymphoprep at RT to each tube.
14. Carefully layer 25 mL of the diluted buffy coat solution onto the 15 mL Lymphoprep layer in each 50 mL conical tube.
  a. Set the pipette controller to the lowest speed setting to avoid mixing the blood with the density gradient.
  b. Tilt the conical tube to approximately 40° and slowly dispense the diluted blood down the inner wall of the tube (Figure 4C).
  c. As the volume increases, gradually return the conical tube to an upright position while continuing to dispense slowly.
    ***Note:*** This helps maintain an interface between the layers.
  d. Make sure that all six conical tubes have 40 mL volume. Top up with PBS if needed.
    **CRITICAL:** Avoid disturbing the Lymphoprep layer by pipetting slowly. A disrupted interface will impair PBMC separation and reduce yield.
15. Centrifuge the conical tubes at 700 × *g* for 30 min at RT using acceleration setting 4 and deceleration setting 1.

**CRITICAL:** Do not use full brake to prevent disruption of the gradient.

16. Using a Pasteur pipette, carefully collect the interphase containing PBMCs from each conical tube (Figures 4D and 4E).
17. Transfer each interphase into an individual 50 mL conical tubes.
18. Fill each conical tube up to 50 mL with PBS supplemented with 2% Fetal Bovine Serum (FBS).
19. Centrifuge at 800 × *g* for 10 min at RT using acceleration setting 9 and deceleration setting 7.
20. Discard the supernatant and resuspend the cell pellets in 5 mL of PBS supplemented with 2% FBS to subsequently pool all resuspended cells into one single 50 mL conical tube.
21. Top up to 50 mL with PBS + 2% FBS and centrifuge at 400 × *g* for 10 min at RT using acceleration setting 9 and deceleration setting 7.
22. Discard the supernatant and resuspend the cell pellet in 50 mL of PBS + 1% FBS at RT and count the cells using a hemocytometer or an automated cell counter.

***Note:*** Due to high cell concentration it is recommended to dilute the cells at least 1:20 in a separate 96-well plate prior to counting.

23. Centrifuge at 400 × *g* for 5 min at RT using acceleration setting 9 and deceleration setting 7.
24. Discard the supernatant and resuspend the pellet in 0.4 mL MACS buffer per 1∗10^8^ cells.
25. Continue isolating CD14^+^ monocytes using CD14 microbeads according to the manufacturer’s instructions for positive selection of CD14^+^ monocytes (Figure 4F).
26. Count the acquired CD14^+^ monocytes using a hemocytometer or an automated cell counter.
27. Resuspend the monocytes at a concentration of 1.25∗10^6^ cells/mL in moDC differentiation culture medium.
28. Plate 12 mL of the cell suspension (15∗10^6^ cells) per T75 cell culture flask.

***Note:*** For culture flasks without a vented filter cap, ensure the cap is unscrewed during incubation to allow adequate gas exchange.

29. Culture the monocytes in the moDC differentiation medium at 37°C in a humidified incubator with 5% CO_2_ for 4-6 days before the planned experimental set-up to allow differentiation into immature moDCs (Figure 4A).

### Establishment of the IEDC co-cultures

**Timing: 4 h, overnight equilibration**

**Figure 5:**
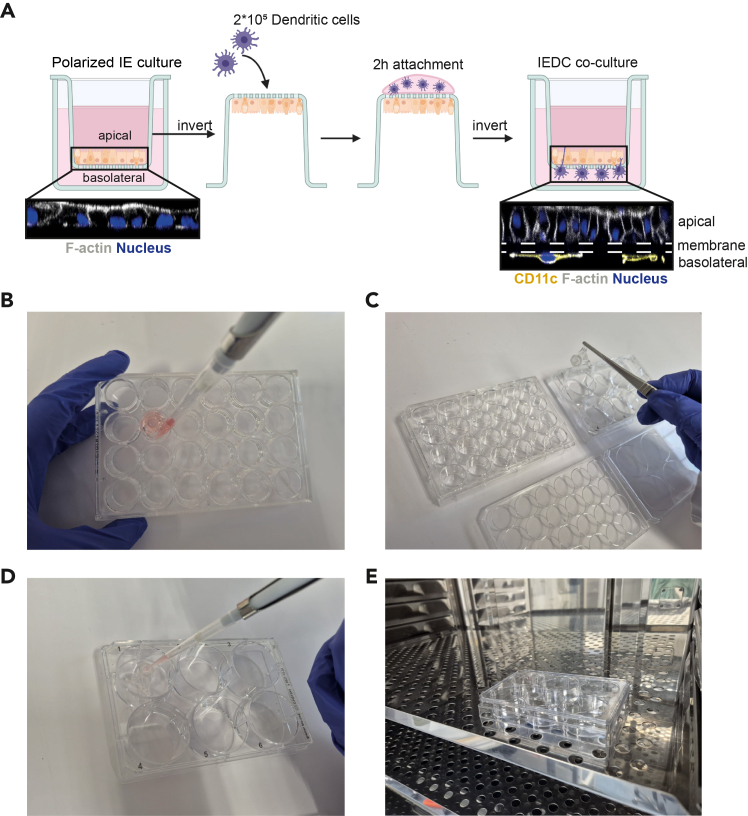
Establishment of intestinal epithelium-dendritic cell co-cultures (A) Schematic overview of the experimental workflow for establishing the intestinal epithelium–DC (IEDC) co-culture model. Confocal z-stack images show intestinal epithelium (IE) culture and IEDC co-culture, with DC surface marker CD11c (yellow), F-actin (grey), nuclei (blue), and the insert membrane indicated by a white dashed line. Figure reprinted and adapted from Rader et al., 2024. (B) Removal of apical and basolateral medium from the intestinal epithelial cell culture insert. (C) Inversion of the insert into a 6-well plate to expose the basolateral membrane. (D) Seeding of 50 μl medium containing 200,000 dendritic cells onto the basolateral side of the insert membrane. (E) Incubation for 2 h to allow dendritic cell adhesion to the basolateral membrane.

In this step, we describe how to establish the IEDC co-culture model combining human intestinal epithelial monolayers with immature monocyte-derived dendritic cells (moDCs) (Figure 5A). We utilized day 4 immature moDCs because of their viability for extended co-culture. Furthermore, they recapitulate key functional properties of intestinal mucosal dendritic cells at steady-state, including luminal sampling and migratory characteristics.

30. On day 4 of differentiation, gently resuspend the moDCs in the culture flask and count the number of cells using a hemocytometer or an automated cell counter.
31. Collect the moDCs in conical tubes and centrifuge at 400 × *g* for 5 min at RT using acceleration setting 9 and deceleration setting 7.
32. Discard the supernatant and resuspend moDCs in pre-warmed IEDC co-culture medium at a concentration of 4∗10^6^ cells/mL.
33. Remove apical and basolateral medium from cell-culture inserts containing the 10-days old intestinal monolayers (Figure 5B).
34. Using sterile forceps, carefully invert the cell-culture inserts into an empty 6-well plate (Figure 5C).

***Optional:*** Instead of 6-well plates, 12-well plates or large Petri dishes may also be used to hold the inverted cell-culture inserts.

***Note:*** Before use, verify that the height of the plate and lid fits properly without touching the basolateral side of the insert membrane.

35. Add 50 μL of the IEDC co-culture medium containing 2∗10^5^ moDCs onto the basolateral (bottom) surface of each inverted insert (Figure 5D).
36. Incubate the inverted inserts in a closed plate at 37°C in a humidified incubator with 5% CO_2_ for 2 h to facilitate moDC attachment to the basolateral surface (Figure 5E).
37. After attachment, carefully revert the inserts to their original orientation into 24-well plates.
38. Add 100 μL IEDC co-culture medium to the apical (insert) side and 600 μL to the basolateral (well) side.
39. Incubate the co-cultures at 37°C for 24 h to allow equilibration of moDCs and intestinal monolayers before downstream applications (see troubleshooting 6).
40. After equilibration, measure TEER to ensure preservation of intestinal epithelial barrier integrity.

***Note:*** Some moDCs may detach during equilibration. After equilibration, refresh the media or transfer inserts to new plates to remove unattached moDCs.

### Viral infection of IEDC co-culture

**Timing: 1 h**

This section describes HIV-1 infection of the IEDC co-cultures for mechanistic and therapeutic studies. In our experiments, multiple HIV-1 variants were used at varying concentrations (between 7.5 × 10^3^ to 1.9 × 10^5^ TCID_50_ per insert) similar conditions can be adapted depending on the virus and experimental aims. Here, the infection protocol is described using the HIV-1 variant NL4.3BaL as an example.

***Note:*** Viral stocks should be produced, quantified, and stored according to established laboratory protocols and institutional biosafety regulations and guidelines.

41. Refresh the apical and basolateral medium of the IEDC co-cultures with IEDC co-culture medium.
42. Add HIV-1 NL4.3BaL at a concentration of 1.87 × 10^5^ TCID_50_ to the apical surface of each insert containing the IEDC co-culture.
43. Incubate the infected co-cultures for 3 days at 37°C in a humidified incubator with 5% CO_2_.

***Optional:*** After 72 h of infection, FD4 permeability can be used to assess barrier integrity in IE cultures only. This assay is not suitable for IEDC co-cultures because dendritic cells take up the fluorescent dextran, confounding the readout.

***Optional:*** Pharmacological agents, biologicals, or other stimuli can be added before or after infection to extend these studies, for example to test antiviral efficacy or modulate epithelial and immune responses.

### Visualization of IEDC co-culture using confocal microscopy

**Timing: 3 days, 2–4 h per day**

This protocol describes the fixation, permeabilization, immunostaining, and mounting of IEDC co-cultures for confocal microscopy. It enables visualization of distinct cell populations, intracellular signaling pathways, and viral markers within the intestinal epithelial-dendritic cell interface.

**Figure 6:**
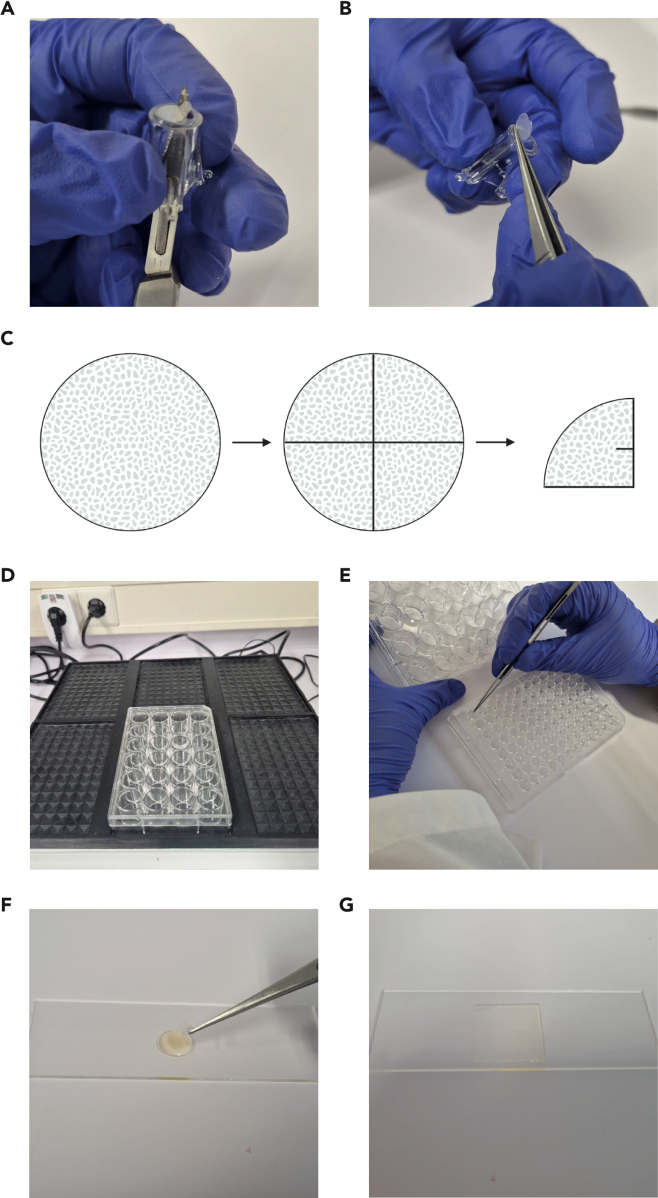
Sample preparation for confocal microscopy (A and B) Removal of the insert membrane containing the cells by carefully cutting out the membrane. (C) Schematic depiction of a quartered insert membrane to allow for multiple separate stainings, a subsequent small incision can be made into the membrane to help identify the apical and basolateral sides of the membrane. (D) Placement of membranes in a 24-well plate, followed by permeabilization, washing, and blocking steps on a shaker. (E) Transfer of samples to a 96-well plate for primary antibody, secondary antibody, and DAPI staining. (F) Mounting of stained samples on glass slides using antifade mounting medium. (G) Placement of a coverslip to allow confocal imaging.
45. For permeabilization, transfer membrane pieces to a 24-well plate containing 500 μL confocal permeabilization buffer (Figure 6D).
  a. Permeabilize samples for 15 min at RT.
  b. Remove the permeabilization buffer.
  c. Wash 3 times with 500 μL PBS on a shaker at 300 rpm.
46. Incubate membrane inserts in blocking solution to reduce nonspecific antibody binding.
  a. Remove all PBS from the wells and add 500 μL fish blocking solution.
  b. Incubate for 60 min at RT on a shaker set to 300 rpm.

***Note:*** Fish blocking solution was selected because it consistently minimized background staining in our hands, alternative blocking reagents were not evaluated in this system.

***Note:*** Membrane pieces are transferred to a 24-well plate to allow storage in a larger volume and to enable staining of different membrane sections at different time points, rather than staining all four sections simultaneously.

44. For the fixation of the IEDC co-cultures:
  a. Remove culture media from both the apical and basolateral sides of the inserts.
  b. Add 200 μL of 4% paraformaldehyde to the apical side and 700 μL of 4% paraformaldehyde (PFA) to the basolateral side.
    ***Note:*** For HIV-1 virus infected samples it is recommended to submerge the complete insert in 4% PFA to make sure all virus is inactivated and fixed. For example, this can be done in a 50 mL conical tube with at least 8 mL 4% PFA per insert.
  c. Incubate for 30 min at room temperature at RT to fix the samples.
    ***Note:*** Thirty min of fixation is required to fully inactivate HIV-1. For non-infected samples 15 min of fixation is sufficient.
  d. Remove the fixative.
  e. Wash 3 times with 100 μL (apical) and 600 μL (basolateral) of PBS at RT.
  f. Using a scalpel, carefully cut the membrane out of the insert, taking care not to damage the cell layer (Figures 6A and 6B).
    ***Optional:*** Cut the membrane insert into 4 pieces to allow multiple staining conditions of the same sample, for example staining for different proteins (Figure 6C).
    ***Note:*** The apical and basolateral sides of the membrane can be difficult to distinguish by eye. To maintain orientation, make a small incision or notch in one corner of each membrane piece while the insert is still in its original position. This will serve as a reference to identify the apical side during subsequent mounting steps (Figure 3C).

**Pause point**: Insert membranes can be stored in the fish blocking solution at 4 °C for up to one week until staining.

47. Primary antibody staining of the insert membrane (Figure 6E).
  a. Dilute primary antibodies in fish serum blocking solution: anti-p24 at 1:100 dilution and anti-CD11c at 1:20 dilution.
  b. Pipette 50 μL of the primary antibody mix per sample into a well of a U-bottom 96-well plate.
  c. Add each membrane quarter sample into the corresponding well containing the antibody mix.
  d. Incubate the plate overnight at 4°C.

**CRITICAL:** Protect sampled from light during and after secondary antibody staining in the next steps to prevent photobleaching of fluorescent dyes.

48. Secondary antibody staining of the insert membrane.
  a. Prepare the secondary antibody mix fresh in blocking solution, keeping it at RT and protected from light until use. The mix consists of anti-mouse-IgG1 Alexa 647 and anti-mouse IgG2b Alexa 546, both at 1:400 dilution, together with the probe phalloidin CruzFlour 488 at 1:1500 dilution to stain F-actin.
  b. Remove the primary antibody mix and wash 3 times with 100 μL PBS on a shaker at 300 RPM at RT.
  c. Add 50 μL of secondary antibody mix to each well.
  d. Incubate for 1 h at RT.
49. DAPI staining of the insert membrane to stain cell nuclei.
  a. Remove the secondary antibody mix and wash 3 times with 100 μL PBS on a shaker at 300 rpm at RT.
  b. Prepare the DAPI working solution according to the manufacturer’s instructions.
  c. Add DAPI working solution and incubate for 5 min at RT on a shaker at 300 RPM RT.
  d. Remove the DAPI solution and wash 3 times with 100 μL PBS on a shaker at 300 RPM at RT.
50. Mounting of the insert membrane on glass slides for confocal imaging.
  a. Pipet 10 μL of ProLong™ Glass Antifade Mountant onto a glass microscopy slide.
  b. Carefully remove the membrane inserts from wells with forceps and gently dry by tapping the membranes on a tissue or filter paper.
  c. If applicable, locate the small incision made on the membrane to identify the apical side.
    ***Note:*** For optimal imaging of the apical membrane cells, place the membrane with the apical side facing up. For imaging of the basolateral side, orient the membrane with the basolateral side facing up.
  d. Place the insert with preferred side up into the ProLong™ Glass Antifade Mountant on the microscope slide (Figure 6F).
  e. Gently place a coverslip on top of the mounted membrane (Figure 6G). Apply slight pressure to the coverslip to eliminate any trapped air beneath the glass.
    ***Note:*** Use slides and coverslips with appropriate dimensions and a thickness compatible with your imaging system.
51. Allow the slides to cure for 24-72 h at RT in the dark to let the mounting medium harden before imaging.
52. Image samples using a confocal microscope (see troubleshooting 7).

**Figure 7:**
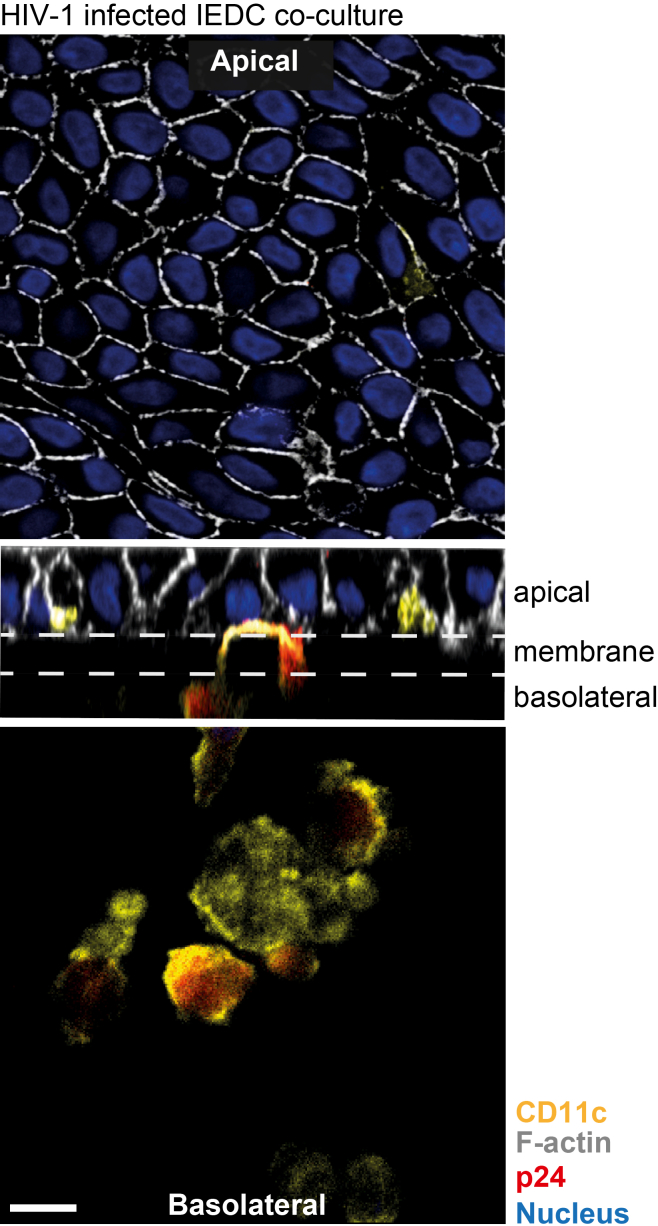
Expected confocal imaging result from HIV-1 infected IEDC co-cultures Representative confocal image of HIV-1 infected IEDC co-culture at 72 h, showing the apical (top panel) and basolateral (bottom panel) sides, and a cross-sectional z-stack view (middle panel). Dendritic cells are visualized by the surface marker CD11c (yellow), F-actin (white), HIV-1 capsid protein p24 (red), and nuclei (blue). Dashed lines indicate the separation of apical and basolateral sides by the insert membrane; scale bar = 10 μm. Figure reprinted and adapted with permission from Rader et al., 2024.^1.^

***Note:*** Staining results example is shown in Figure 7.

### Flow cytometry analyses to immunophenotype basolaterally harvested dendritic cells from IEDC co-cultures

**Timing: 3 h**

This section describes harvesting, fixation, permeabilization, and immunophenotyping of basolateral dendritic cells detached from infected IEDC co-cultures for multiparameter flow cytometry analysis. The cells are stained using combinations of DC lineage markers (CD11c, DC-SIGN) and activation/maturation markers (CD83/CD86) to characterize DCs.

53. Harvesting of basolateral DCs (see troubleshooting 8).
  a. Seventy-two h post-infection with HIV-1 NL4.3BaL, remove the medium from both the apical and basolateral sides of the inserts.
  b. Wash both sides once with pre-warmed PBS (200 μl apical, 600 μl basolateral).
  c. Add 300 μL Accutase Cell Detachment Solution to the basolateral side.
  d. Incubate at 37°C in a humidified incubator with 5% CO_2_ for 10 min to detach basolateral DCs.
  e. With the cell culture insert still placed inside the well, gently aspirate and dispense the suspension in the well several times to ensure detachment of all basolateral cells.
  f. Transfer the cell suspension to a 1.5 mL Eppendorf tube.
54. Fixation of the harvested cells.
  a. Fix the harvested cells by adding 4% PFA and incubating them at RT for 30 min.
  b. Wash the fixed cells once by resuspending them in 200 μl PBS, then centrifuge at 400 × *g* for 5 min.

**Pause point**: Fixed cells can be stored in PBS at 4 °C for up to one week until staining.

***Note:*** Fixed cells from a single culture insert can be divided into two tubes for separate flow cytometry analyses.

**Figure 8:**
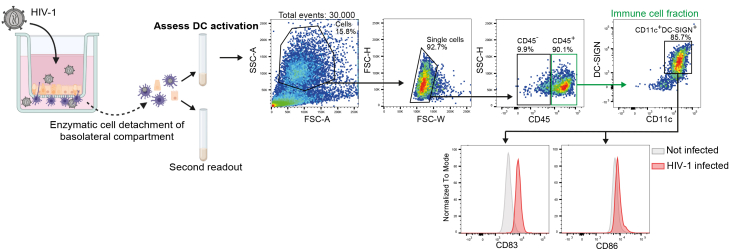
Expected flow cytometry results of dendritic cells detached from IEDC co-cultures 72 h post HIV-1 infection Schematic representation of enzymatic detachment of cells from the basolateral side of IEDC co-culture inserts. Cells recovered from a single insert can be divided into two tubes for separate flow cytometric analyses. Gating strategy to show the percentage of non-immune epithelial cell fraction (CD45^-^), total immune cell fraction (CD45^+^), and dendritic cells (CD45^+^CD11c^+^DC-SIGN^+^) recovered from IEDC co-cultures. Histograms depict the expression of canonical activation markers CD83 and CD86 on CD45^+^CD11c^+^DC-SIGN^+^ dendritic cells upon HIV-1 infection compared with uninfected controls. Figure reprinted and adapted with permission from Rader et al., 2024.^1^

***Note:*** During the Accutase-based harvesting at the basolateral side, the epithelial barrier may partially lose integrity, causing some epithelial cells (CD45^-^) to detach, be co-harvested with basolateral DCs, and subsequently be detected and distinguished by multiparameter flow cytometry.

55. Intracellular and surface staining cells.
  a. Transfer the cells in PBS from the Eppendorf tube to a V-bottom 96-well plate, and centrifuge the plate at 400 × *g* for 5 min to pellet the cells.
  b. Remove supernatant and wash the fixed cells once with PBS supplemented with 0.5% saponin and 1% BSA by centrifugation at 400 × *g* for 5 min at RT.
  c. Prepare the following antibody cocktail in PBS with 0.5% saponin and 1% BSA: anti-CD45-BV711 at 1:100 dilution, CD11c-PE-Cy7 at 1:50 dilution, anti-DC-SIGN-AF750 at 1:100 dilution, CD83-AF647 at 1:50 dilution, and CD86-AF488 at 1:100.
  d. Resuspend cells in 50 μL of the antibody mix and incubate for 30 min at 4°C on a plate shaker at 250 rpm, protected from light.
  e. Wash the stained cells with PBS by centrifugation at 400 × *g* for 5 min at RT.
  f. Resuspend cells in PBS for acquisition.
  g. Measure samples on a flow cytometer such as the BD FACSymphony A1. Gating strategy is depicted in Figure 8.

***Optional:*** Other markers can also be used in this protocol to assess HIV-1 infection and specific DC phenotype and functionality, as demonstrated in the associated article.^1^

## Expected outcomes

This protocol yields polarized, differentiated 2D IE cultures derived from human fetal intestinal organoids. After 10 days of culture on collagen-coated inserts, IE cultures typically reach TEER values above 200 Ω·cm^2^, consistent with the formation of an epithelial barrier, as further confirmed by paracellular permeability assays. Co-culture with moDCs establishes IEDC co-cultures that provide a human mucosal model with experimental access to both apical and basolateral compartments. This configuration enables apical or basolateral HIV-1 exposure, application of biologicals or drugs to defined compartments, and longitudinal sampling of soluble factors. Confocal microscopy of IEDC co-cultures allows visualization of epithelial organization, moDC localization. and the spatial arrangement of cells at the epithelial-dendritic cell interface. In parallel, multiparameter flow cytometry of harvested basolateral cell fractions enables profiling of moDC phenotype and immune activation. Together, these complementary readouts support mechanistic investigation of mucosal barrier dynamics and dendritic cell activation within a controlled human intestinal infection model, and provide a versatile platform to study HIV-1 entry and intracellular trafficking and host signaling pathways, as demonstrated in the associated study by Rader et al.^1^

## Limitations

The use of fetal-derived small intestinal organoids is an important consideration when interpreting results obtained with this model. These organoids recapitulate key intestinal epithelial lineages and display barrier-forming capacity in vitro, making them a suitable model for studying intestinal barrier function and mucosal HIV-1 infection.^12^^,^^13^ They also offer robust expansion and experimental reproducibility. However, epithelial maturation and immune regulation pathways continue to evolve postnatally; therefore, validation in adult-derived organoids would further strengthen the physiological relevance of the findings.

Primary organoids also preserve donor-specific genetic variability, enabling investigation of how host background influences epithelial barrier properties and HIV-1-epithelium interactions. A limitation of the IEDC co-culture is that it uses allogeneic moDCs from peripheral blood rather than autologous, tissue-resident intestinal DCs, because primary intestinal DCs are scarce and difficult to obtain in sufficient numbers. HLA mismatching between epithelial and DC donors may introduce allogeneic immune activation. In this study, this limitation was partially addressed by including multiple independent donor combinations and focusing on reproducible patterns across donors.^1^ Further iterations incorporating adult- and patient-derived organoids together with autologous immune cells could enable precision-medicine studies of mucosal HIV-1 acquisition.

Finally, this protocol uses a static 2D co-culture without perfusion. Future integration of IEDC co-cultures into microphysiological gut-on-chip platforms with controlled luminal and basolateral flow, as well as oxygen gradients, could enhance physiological relevance. Such systems would enable studies of shear stress–dependent barrier regulation, dynamic epithelial–immune interactions, and the impact of flow and oxygen tension on HIV-1 infection kinetics and barrier injury and repair.

## Troubleshooting

### Problem 1

Insufficient cells obtained from 3D organoids (Note step 1).

### Potential solution

Extend the expansion of 3D organoid until higher density is reached before initiating 2D monolayer formation. In our hands, organoids which have been passaged over 25 times grow slower and may need to be replaced by earlier passage cultures to prevent insufficient cell counts.

### Problem 2

Intestinal epithelial cells do not form a confluent monolayer on collagen-coated inserts (step 4).

### Potential solution

Collagen coating is likely suboptimal due to incorrect concentration or an old/faulty batch. If needed, check batch concentrations or use a new collagen batch.

### Problem 3

TEER values remain low and do not increase over time (Note step 9).

### Potential solution

Increase the seeding density. We have successfully improved barrier formation by increasing the seeding density in increments of 10.000 cells per inserts up to 150.000 cells total for slow growing cells.

### Problem 4

TEER values decrease after initially reaching confluency (Note step 9).

### Potential solution

Monolayers reach confluency too rapidly during the expansion phase, leading to barrier deterioration over time. Reduce the initial seeding density in steps of 10.000 cells per insert (down to a minimum of 50.000 cells), or shorten the expansion phase from 5 days to 4 or 3 days before switching to differentiation medium.

### Problem 5

Limited access to fresh primary human blood for moDC generation (step 12).

### Potential solution

Cryopreserve PBMCs and isolate monocytes on the day of the experiment. PBMCs can be frozen in 10% DMSO in antibiotic-free RPMI supplemented with 30% FBS and thawed when required to generate differentiated immature moDCs.

### Problem 6

Dendritic cells fail to attach to the basolateral side of the insert (step 39).

### Potential solution

Assess moDC viability prior to attachment using a viability dye, such as Trypan Blue, during counting and adjust the number of viable cells accordingly. Ensure than handling during isolation and culture supports high viability.

### Problem 7

High membrane autofluorescence or background signal in confocal imaging (step 52).

### Potential solution

Use inserts suitable for imaging with low autofluorescence. In our experiment, we used CellQart clear inserts specifically designed for microscopy.

### Problem 8

Impaired retrieval of basolateral DCs from IEDC co-cultures for flow cytometry (step 54).

### Potential solution

This is likely caused by low initial moDC viability or unintended maturation impairing initial attachment. Confirm viability as described in trouble shooting step 6. And ensure moDCs are immature prior to seeding, which can be determined by flow cytometry (CD83/CD86 markers) and by microscopy where dendrite formation and clumping suggests maturation.

## Resource availability

### Lead contact

Further information and requests for resources and reagents should be directed to and will be fulfilled by the lead contact, Carla Mónica Sampaio Ribeiro (c.m.ribeiro@amsterdamumc.nl).

### Technical contact

Technical questions on executing this protocol should be directed to and will be answered by the technical contact, Carla Mónica Sampaio Ribeiro (c.m.ribeiro@amsterdamumc.nl).

### Materials availability

This study did not generate new unique reagents.

### Data and code availability

All data presented are derived from the same experiments as reported in Rader et al.^1^ The microscopy and flow cytometry datasets supporting this study have not been deposited in a public repository but are available from the lead author upon request, provided that the data will be used within the scope of the originally provided informed consent.

## Acknowledgments

This work was supported by an 10.13039/100019573Amsterdam UMC grant and funding from the 10.13039/501100003246Dutch Research Council (10.13039/501100003246NWO-ZonMw) through VIDI and ASPASIA grants under grant agreements 91718331 and 015.014.030, respectively. The authors thank John L. van Hamme, Tanja M. Kaptein, and Esther M. Zijlstra-Willems from Amsterdam UMC for their assistance in acquiring the PBMC isolation images. We also thank Dr. Renée Schreurs, Dr. Vanesa Muncan, and Dr. Lance Mulder from Amsterdam UMC for sharing protocols for epithelial monolayer culture and confocal microscopy sample preparation. The graphical illustrations were created with BioRender.com (https://biorender.com) under an academic subscription.

## Author contributions

A.G.R. conceived and optimized all experimental protocols, performed experiments, collected and analyzed the data, and wrote the initial and revised versions of the manuscript. C.M.S.R. supervised the study, contributed to protocol development and study design, and revised both versions of the manuscript.

## Declaration of interests

The authors declare no competing interests.

## References

[1] Rader A.G., Cloherty A.P.M., Patel K.S., Almandawi D.D.A., Pajkrt D., Wolthers K.C., Sridhar A., van Piggelen S., Baaij L.E., Schreurs R.R.C.E., Ribeiro C.M.S. (2024). HIV-1 exploits LBPA-dependent intraepithelial trafficking for productive infection of human intestinal mucosa. PLoS Pathog..

[2] Coleman C.M., Wu L. (2009). HIV interactions with monocytes and dendritic cells: viral latency and reservoirs. Retrovirology.

[3] Manches O., Frleta D., Bhardwaj N. (2014). Dendritic cells in progression and pathology of HIV infection. Trends Immunol..

[4] Cavarelli M., Foglieni C., Rescigno M., Scarlatti G. (2013). R5 HIV-1 envelope attracts dendritic cells to cross the human intestinal epithelium and sample luminal virions via engagement of the CCR5. EMBO Mol. Med..

[5] Kämpfer A.A.M., Urbán P., Gioria S., Kanase N., Stone V., Kinsner-Ovaskainen A. (2017). Development of an in vitro co-culture model to mimic the human intestine in healthy and diseased state. Toxicol. Vitro.

[6] Liu Y., Chen Y.G. (2018). 2D- and 3D-Based Intestinal Stem Cell Cultures for Personalized Medicine. Cells.

[7] Ahn J., Jung K.B., Kwon O., Choi M.S., Ahn J.H., Han H.Y., Jung C.R., Yoon S., Son M.Y., Oh J.H. (2021). Impedance Measurement System for Assessing the Barrier Integrity of Three-Dimensional Human Intestinal Organoids. Anal. Chem..

[8] Bardenbacher M., Ruder B., Britzen-Laurent N., Schmid B., Waldner M., Naschberger E., Scharl M., Müller W., Günther C., Becker C. (2019). Permeability analyses and three dimensional imaging of interferon gamma-induced barrier disintegration in intestinal organoids. Stem Cell Res..

[9] Schreurs R.R.C.E., Baumdick M.E., Drewniak A., Bunders M.J. (2021). In vitro co-culture of human intestinal organoids and lamina propria-derived CD4(+) T cells. STAR Protoc..

[10] Cloherty A.P.M., van Teijlingen N.H., Eisden T.J.T.H.D., van Hamme J.L., Rader A.G., Geijtenbeek T.B.H., Schreurs R.R.C.E., Ribeiro C.M.S. (2021). Autophagy-enhancing drugs limit mucosal HIV-1 acquisition and suppress viral replication ex vivo. Sci. Rep..

[11] van Smoorenburg M.Y., Remmerswaal E.B.M., Segui-Perez C., van Hamme J.L., Strijbis K., Geijtenbeek T.B.H. (2025). Vaginal Prevotella timonensis Bacteria Enhance HIV-1 Uptake and Differentially Affect Transmission by Distinct Primary Dendritic Cell Subsets. Eur. J. Immunol..

[12] Roodsant T., Navis M., Aknouch I., Renes I.B., van Elburg R.M., Pajkrt D., Wolthers K.C., Schultsz C., van der Ark K.C.H., Sridhar A., Muncan V. (2020). A Human 2D Primary Organoid-Derived Epithelial Monolayer Model to Study Host-Pathogen Interaction in the Small Intestine. Front. Cell. Infect. Microbiol..

[13] Gómez D.P., Boudreau F. (2021). Organoids and Their Use in Modeling Gut Epithelial Cell Lineage Differentiation and Barrier Properties During Intestinal Diseases. Front. Cell Dev. Biol..

